# Psychosocial work conditions and prediabetes risks: a cross-sectional study in middle-aged men and women

**DOI:** 10.1038/s41598-023-28420-7

**Published:** 2023-01-21

**Authors:** C. Schmidt, A. Gummesson, F. Bäckhed, Göran Bergström, M. Söderberg

**Affiliations:** 1 Wallenberg Laboratory for Cardiovascular Research, Department of Molecular and Clinical Medicine, Institute of Medicine, Sahlgrenska Academy, University of Gothenburg, 413 45, Gothenburg, Sweden; 2 Department of Clinical Genetics and Genomics, Sahlgrenska University Hospital, Gothenburg, Sweden; 3 Department of Clinical Physiology, Sahlgrenska University Hospital, Gothenburg, Sweden; 4 Occupational and Environmental Medicine, School of Public Health and Community Medicine, Institute of Medicine, Sahlgrenska Academy, University of Gothenburg, Gothenburg, Sweden

**Keywords:** Diseases, Endocrinology

## Abstract

Prediabetes is a condition between diabetes and normoglycemia, and is a state of major health concern, as a large proportion of people with prediabetes are likely to develop diabetes which is associated with high mortality and morbidity. The purpose of this study was to investigate whether adverse psychosocial work conditions, based on the Job Demand-Control-social support model, increases risk for early dysregulated glucose metabolism in 50–64-year-old men and women. Job conditions were measured with the Swedish Demand-Control-Support questionnaire. Impaired glucose metabolism was assessed by an oral glucose tolerance test. Differences between groups were analyzed with Chi-square test and one-way ANOVA with Bonferroni post-hoc test. Odds ratios (OR) and 95% confidence intervals (95% CI) between Job Demand-control-support and prediabetes outcome were calculated with multiple logistic regression. Results from an adjusted logistic regression model showed that in men and woman separately, an active work situation (high demands-high control) was associated with significantly lower prediabetes risk (OR 0.657, 95% CI 0.513–0.842). This finding is consistent through all logistic regression models with different levels of adjustments. Further, the current study does not lend support for the hypothesis that work conditions characterized by high demands-low control were associated with dysregulated glucose metabolism in men nor women despite accumulation of many life-style related risk factors in the high strain group. In conclusion, we could show that men and women assessing their work conditions as active, had lower risk for prediabetes.

## Introduction

Prediabetes is a state between diabetes and normoglycemia, in which the body produces more insulin than normal to regulate elevated plasma glucose levels. Prediabetes is a health concern that carries a high risk of developing into type 2 diabetes mellitus (T2DM) and its high risk of morbidity and mortality^1^. The condition is largely asymptomatic and includes individuals with impaired fasting glucose (IFG) and impaired glucose tolerance (IGT), as well as a combination of IFG and IGT, called combined glucose intolerance (CGI)^2–4^. Lifestyle plays a significant role in development of prediabetes and T2DM^5^

Our modern lifestyle is lived at a breakneck speed where family-, social- high work demands easily can overpower time and resources. This may cause both physical and emotional stress that can take great toll on health and may also affect glucose homeostasis^6^. One of the most evaluated models for measuring stressful working conditions is the Job Demand-Control-social support (JDCS) model. In this model construct, the “demand” variable captures psychological workload, while “control” measures the employee’s influence over the content and volume of work tasks. Commonly, these two variables are dichotomized into high/low and combined to form four different categories of work environments, high strain (high demand-low control), active (high demand-high control), passive (low demand-low control) and low-strain (low demand-high control)^7^. Further, the combination of high-strain and low social support at work has been referred to as “iso-strain”. Social support from work colleagues or managers is expected to be health-promoting, while lack of social support has emerged as a risk factor for ill-health outcomes^8^. While high strain working conditions has been associated with development of T2DM in several studies^9^, much less is known about the early phases of the disease and only a limited number of studies have addressed high strain in prediabetes^10,11^. These two studies use HbA1c levels to suggest an association between high strain and prediabetes. There are to our knowledge no studies using the standard 2-h oral glucose tolerance test (OGTT) to define early glucose dysregulation^12^. Further, no studies have used HOMA-IR levels in association with high strain.

The primary aim of this study was to investigate whether adverse psychosocial work conditions evaluated with the Job Demand-Control-social support model, increases risk for early dysregulated glucose metabolism in 50–64-year-old men and women. We also evaluated the association of disturbed glucose metabolism with other combinations of work-related demand and control.

## Material and methods

### Study population

The study sample comprises the pooled participants from the “Microbiota, development of type 2 diabetes and cardiovascular disease study” (IGT-study) and a sub-cohort from the Swedish CArdioPulmonary BioImage Study (SCAPIS) i.e. SCAPIS-Gothenburg. In both studies, results from fasting capillary glucose and an oral glucose tolerance test (OGTT) were used to categorize each individual’s glucose homeostasis using the WHO methodology and criteria^12^. From these two studies, the following subgroups were included in the current study: normal glucose tolerance (NGT), impaired fasting glucose (IFG), impaired glucose tolerance (IGT) and combined glucose intolerance (CGI).

#### Original study 1: Microbiota, development of type 2 diabetes and cardiovascular disease study

The study objective was to investigate if the gut microbiota is related with to future risk of diabetes and atherosclerosis. The ethics committee at Gothenburg University approved the study (Dnr 560-13) which was conducted in accordance with the Declaration of Helsinki.

Men and women, born in Sweden and aged 50–64 years were randomly selected from the Swedish population registry. Initial contact was made by sending out an information brochure asking the recipient to contact the research group for a screening visit.

Inclusion criteria in the IGT-study were fulfilled if the participants had fasting glucose and 2-h glucose values corresponding to T2DM, IFG, IGT or CGI. Participants were also included if they showed increased risk for future diabetes according to the Finnish Diabetes Risk Score (FINDRISC, score > 14)^13^ or had 2 first degree relatives with T2DM. Participants with NGT and no increased risk of developing T2DM according to the FINDRISC questionnaire (≤ 14) were randomized (1:4) to inclusion.

Exclusion criteria were: known diabetes, other severe disease that may jeopardize interpretation of results for example Mb Chron’s, inflammatory bowel disease rheumatic diseases or treatment with steroids or immune-modulating treatment, cancer (unless no relapse during 5 years of follow-up), pharmacological treatment of infection during last 3 months and major cognitive dysfunction.

The study recruited 5191 eligible participants, of which 1965 participants were included in the IGT-study, in the current study, only participants with IFG, IGT, CGI and NGT were eligible for inclusion.

#### Original study 2: The Swedish CArdioPulmonary BioImage Study (SCAPIS)-Gothenburg

The study recruited 30,154 participants aged 50–64 years and examinations were performed at six Swedish university hospitals (in Gothenburg, Linköping, Malmö/Lund, Stockholm, Umeå and Uppsala)^14^.

SCAPIS has been approved as a multicentre trial by the ethics committee at Umea University (Dnr 2010/228-31) and was conducted in accordance with the Declaration of Helsinki.

The recruitment procedure has been described previously^14^. In brief, men and women aged 50–64 years were randomly selected from the Swedish population registry. An appointment was arranged at the study centre if contact was made and the recipient agreed to take part. No exclusion criteria were applied except the inability to understand written and spoken Swedish for informed consent. A total of 6265 participants were recruited at the Gothenburg study centre of which 3346 participants without known T2DM also performed an OGTT. All participants with valid data on fasting glucose and 2-h glucose and fulfilling criteria for NGT, IFG, IGT or CGI were eligible for the current study (n = 3322).

### Informed consent

All participants in the studies received both written and oral information before they gave their written consent to participate.

The two studies followed similar protocols and methodology to facilitate pooling.

### Anthropometry and blood pressure

Weight was measured to the nearest 0.1 kg on digital SECA 910 electronic scales (Vogel and Halke, Hamburg, Germany), with the participants in light clothing. Body height, waist- and hip circumference were measured according to current recommendations^15^. Systolic- and diastolic blood pressures (SBP and DBP) were measured twice with an automatic device (Omron M10‐IT, Omron Health care Co, Kyoto, Japan) and the mean of the measurements used.

### Questionnaires

A questionnaire, containing 140 questions separated into sets relating to factors central to the research aims, was used to collect detailed information on self‐reported health, family history, medication, occupational and environmental exposure, lifestyle, leisure-time physical activity (LTPA), psychosocial well‐being, socioeconomic status and other social determinants.

Further, to calculate the risk for future diabetes in each participant, the FINDRISC questionnaire was administered which captures clinical characteristics related to development of T2DM: BMI; waist circumference; physical activity; dietary consumption of fruits, vegetables, and berries; use of antihypertensive medication; history of high blood glucose and family history of diabetes^13^.

### Job Demand-Control-social support questionnaire

Psychosocial work conditions were recorded in an additional questionnaire, which focused on work environment aspects. Job Demand-Control-social support was measured with the Swedish Demand-Control-Support questionnaire^16^. According to standard procedures, all items were positively inverted so that a high score equated either high demands, high control or high social support, and then added up separately. Since using sum scores, participants completely lacking filled-in items or lacking ≥ 50% filled-in items for either demand or control, were excluded (n = 1287). Data on participants with < 50% missing items were imputed using multiple imputation. The sum scores of demand, control and social support were dichotomized into high or low by the median values of the distributions and combined into: high strain (high demand-low control), active (high demand-high control), passive (low demand-low control) and low strain (low demand-high control). ISO-strain was defined as the combination of high demands-low control-low social support.

### Biochemistry and biochemical sample storage

A venous blood sample (100 mL) was collected from participants after an overnight fast and is used for immediate analysis and stored in a biobank for later analysis (cholesterol, HDL, triglycerides, measured LDL, HbA1c, high-sensitivity C-reactive protein and creatinine). Capillary glucose in the fasting state and 2 h after an oral glucose tolerance test (OGTT, 75 g) was used to categorize each individual’s glucose homeostasis using the WHO criteria^12^. If fasting glucose or 2-h glucose suggested T2DM diagnosis, a repeat sample was taken at the second visit to establish a potential diagnosis of diabetes^12,17^. Homeostatic Model Assessment of Insulin Resistance (HOMA-IR) was calculated using the formula: HOMA-IR = [glucose (nmol/L) * insulin (μU/mL)/22.5], using fasting values^18^.

### Statistical analyses

The analyses were performed by the statistical programs IBM SPSS Statistics (version 27) or SAS statistical software (version 15.1 for Windows). Differences between groups were analyzed with Chi-square test for categorical variables and one-way ANOVA with Bonferroni post-hoc test for continuous variables, using the low strain group as reference.

Odds ratios (OR) and 95% confidence intervals (95% CI) between Job Demand-Control-social support and prediabetes outcome were calculated with multiple logistic regression. In the analyses JDCS variables were analyzed as categorical variables; high strain, active, passive and low strain, using low strain as reference. In analyses investigating ISO-strain low strain-high support was used as a reference. A 95% CI not including 1 or a *p*-value < 0.05 were considered as statistically significant. Categorical and continuous variables are presented as frequencies and percent and as mean and standard deviations (SDs), respectively.

Several potential covariates were considered, which were entered stepwise in three models. The first model only included age, education, smoking, alcohol consumption and physical exercise. In the second model, diabetes heredity, metabolic syndrome and body-mass index (BMI) were added to the same covariates as in the first model. Age, alcohol consumption, and BMI were entered as continuous variables in years (age), in grams per day (alcohol) or in centimetres (waist circumference). Smoking was analysed as current smoker, ex-smoker and never-smoker (= reference). Education was entered categorically as no education, elementary education, secondary education or high education (= reference). Physical exercise was entered categorically as: “Sedentary. Being almost completely inactive: reading, TV watching, movies, using computers or doing other sedentary activities during leisure time”, “Some physical activity during at least 4 h/week as riding a bicycle or walking to work, walking or skiing with the family, gardening, fishing, table tennis, bowling, etc.” “Regular physical activity and training (moderate PA) such as heavy gardening, running, swimming, calisthenics, tennis, badminton and similar activities for at least 2–3 h/week”. “Regular hard physical training for competition sports (vigorous PA): running events, orienteering, skiing, swimming, soccer, racing, European handboll, etc. Several times per week.” Diabetes heredity was measured with one self-reported survey question, “Do your father, mother, siblings or children have diabetes?” (yes/no). The participants were classified as having metabolic syndrome or not, based on the presence of any three out of five parameters. The five parameters were: increased waist circumference (≥ 88 cm in women; ≥ 102 cm in men): increased triglycerides (≥ 1.7 mmol/l or drug treatment for elevated triglycerides) reduced HDL cholesterol (< 1.3 mmol/l in women; < 1.03 mmol/l in men) or treatment with statins, elevated blood pressure (SBP ≥ 130 and/or DBP ≥ 85) or hypertensive drug treatment, elevated fasting glucose (≥ 5.5 mmol/l) or treatment with antidiabetic drugs or insulin.

## Results

### Descriptive findings

From a total number of participants of 5287 participants in both studies combined, the number of eligible participants with impaired- or normal glucose metabolism who had complete data from the OGTT and from the questionnaire on psychosocial work conditions were 3322 individuals (IGT Microbiota (n = 1301), SCAPIS (n = 2021)). The combined cohort consisted of 911 participants with prediabetes (IFG = 287, IGT = 469 and CGI = 155) and 2411 participants with NGT. Slightly more than half of the participants (51%) in the study population were women, 74% were married or cohabitated, 47% of the participants had a university degree and 95% were employed (Table 1).

**Table 1:** Characteristics of the study population and the different groups of psychosocial work conditions.

Variables	All (n = 3322)	Low strain (n = 851) (reference)	Passive (n = 872)	Active (n = 817)	High strain (n = 782)
Women [n, (%)]	1707 (51.4)	365 (42.9)	452 (51.8)***	402 (49.2)*	488 (62.4)***
Age (years)	57.6 ± 4.3	57.4 ± 4.3	57.4 ± 4.3	57.1 ± 4.1*	56.9 ± 4.2**
Marital status [n, (%)]					
Married or cohabitated	2462 (74.1)	659 (77.8)	627 (72.0)**	647 (79.4)	529 (68.4)***
Single (unmarried, divorced, widowed)	844 (25.3)	188 (25.5)	244 (22.2)	188 (28.0)	244 (31.6)
University degree [n, (%)]	1576 (47.4)	465 (54.9)	293 (33.7)***	523 (64.2)***	295 (38.2)***
Employment [n, (%)]	3161 (95.2)	812 (95.8)	833 (95.6)	793 (97.3)	723 (93.3)
Long-term sickness absence [n, (%)]	64 (1.9)	6 (0.7)	11 (1.3)	11 (1.3)	36 (4.6)***
Support at work from colleagues [n (%)]					
Always	447 (13.5)	151 (18.1)	118 (13.5)	95 (11.6)	83 (10.6)*
Support at work from nearest superior [n, (%)]					
Always/Often	1177 (35.4)	373 (43.8)	308 (35.3)	274 (33.5)	222 (28.4)***
Active smoker [n, (%)]	284 (8.7)	67 (8.0)	76 (8.8)	51 (6.3)	90 (11.7)*
Alcohol consumption (g/day)	6.2 ± 6.7	6.8 ± 7.3	6.0 ± 6.6	6.3 ± 6.2	5.6 ± 6.7**
Regular physical exercise [n, (%)]	402 (12.1)	117 (13.8)	97 (11.2)	115 (14.2)	73 (9.4)**
Body Mass Index	26.8 ± 4.2	26.7 ± 4.0	26.7 ± 4.1	26.6 ± 4.1	27.2 ± 4.5*
Abdominal obesity^a^ [n, (%)]	1411 (42.5)	341 (40.1)	373 (42.9)	310 (37.9)	387 (49.5)***
General obesity^b^ [n, (%)]	672 (20.2)	158 (18.6)	178 (20.4)	152 (18.6)	184 (23.5)*
Blood pressure (mmHg)					
Systolic	123 ± 16	123 ± 16	124 ± 16	121 ± 16	123 ± 17
Diastolic	75 ± 11	75 ± 10	76 ± 11	74 ± 11	76 ± 11
High blood pressure^c^ [n, (%)]	1356 (40.8)	338 (39.7)	380 (43.6)	302 (37.9)	336 (43.0)
Metabolic Syndrome according to NCEP [n, (%)]	468 (14.1)	124 (14.7)	132 (14.8)	115 (13.8)	131 (16.9)
Family history of diabetes [n, (%)]	1160 (34.9)	270 (31.8)	300 (34.4)	287 (35.3)	303 (39.1)**
Prediabetes [n, (%)]					
IGT	287 (8.6)	82 (9.6)	81 (9.3)	55 (6.7)*	69 (8.8)
IFG	469 (14.1)	143 (16.8)	112 (12.8)*	96 (11.8)**	118 (15.2)
CGI	155 (4.7)	48 (5.6)	42 (4.8)	38 (4.7)	27 (3.5)*
NGT	2411 (72.6)	479 (56.3)	514 (58.0)	506 (61.9)*	433 (55.4)
HbA1c (mmol/mol)	34.6 ± 3.4	34.6 ± 3.5	34.8 ± 3.6	34.5 ± 3.2	34.7 ± 3.4
HOMA-IR^e^	1.33 ± 0.93	1.31 ± 0.91	1.32 ± 0.91	1.33 ± 0.92	1.37 ± 1.0
Triglycerides mmol/L	1.19 ± 0.75	1.20 ± 0.71	1.17 ± 0.61	1.18 ± 0.75	1.22 ± 0.91
High triglycerides^d^ [n, (%)]	556 (16.7)	148 (17.5)	141 (16.2)	132 (16.2)	135 (17.4)
HDL-C mmol/L	1.71 ± 0.49	1.70 ± 0.51	1.70 ± 0.49	1.70 ± 0.49	1.74 ± 0.48
Low HDL[n, (%)]^f^	56 (1.7)	7 (0.8)	14 (1.6)	18 (2.2)*	17 (2.2)*
hsCRP^g^	1.8 ± 3.4	1.6 ± 2.0	1.7 ± 2.6	1.8 ± 4.8	2.0 ± 3.6*

IGT, impaired glucose tolerance; IFG, impaired fasting glucose tolerance; CGI, combined impaired glucose tolerance; NGT, normal glucose tolerance.

***p < 0.0001, **p < 0.001, *p < 0.05 compared with low strain.

^a^Waist circumference > 102 cm in men and > 88 cm in women.

^b^Body mass index ≥ 30 kg/m^2^.

^c^Systolic blood pressure >  = 130 mmHg and/or diastolic blood pressure >  = 85 mmHg or medication.

^d^Triglycerides >  = 1.7 mmol/L.

^e^HOMA-IR; Homeostatic Model Assessment for Insulin Resistance.

^f^HDL-cholesterol < 0.9 mmol/L in men and < 1.1 mmol/L in women.

^g^hsCRP; high-sensitivity C-reactive protein.

Compared to the reference group (low strain), the high strain group was characterized by a larger number of women, being slightly younger, fewer being married or cohabitated and a smaller number of participants with a university degree (Table 1). Further, this group showed a more unfavourable lifestyle- and metabolic health profile with a larger number of active smokers, more participants with abdominal- and general obesity and increased BMI, as well as more participants having low HDL-cholesterol levels, and somewhat increased high-sensitivity C-reactive protein levels (hsCRP) compared to the low strain group. Further, the high strain group consumed less alcohol (g/day) and was less likely to engage in physical activity.

Moreover, the high strain group showed increased sick leave, and assessed that the support from colleagues as well as from immediate superior at work was significantly lower compared to the low strain group.

The high strain group also had less participants with CGI, but showed a higher number of participants with a family history of diabetes, compared to the reference group.

The groups assessing their work strain as active or passive, were characterized by an increased number of women and while the active group had more participants with a university degree, the passive group had less participants with university degree and fewer in this group were married or cohabitated. Further, a smaller number of participants in the active- and passive- strain groups were found in the IFG group, and for the active group, less participants were diagnosed with IGT, and more participants showed a normal glucose tolerance. Also, the active work group showed a greater number of participants with low HDL values. In addition, a significant increasing trend from low-strain to high-strain group was observed for diabetes heredity (*p* < 0.001), however, no difference was observed in HbA1c values between the groups. The HOMA-IR values were not increased according to established cut-offs^18^. No differences were observed in HOMA-IR values between the groups, though a non-significant linear trend was noticed with lower values in the low strain and the highest values in the high strain group.

### Association between work conditions and prediabetes

The logistic regression analysis models performed for risk of prediabetes are shown in Table 2.

**Table 2:** Logistic regression job demand-control-social support and pre-diabetes (IGT + IFG + CGI) (n = 3322).

	All				Men				Women			
	Cases	OR	95% CI		Cases	OR	95% CI		Cases	OR	95% CI	
Model 1												
High strain	213	0.853	0.675	1.077	88	0.841	0.599	1.180	125	0.912	0.651	1.278
Active	186	**0.719**	**0.570**	**0.908**	98	**0.674**	**0.493**	**0.922**	88	0.775	0.546	1.102
Passive	228	**0.753**	**0.600**	**0.946**	121	**0.727**	**0.536**	**0.987**	107	0.808	0.572	1.142
Low strain (ref)	262	1			161	1			101	1		
ISO-strain	154	0.803	0.602	1.071	63	0.875	0.579	1.322	91	0.764	0.500	1.165
Low strain-high support (ref)	177	1			106	1			71	1		
Model 2												
High strain	213	0.802	0.626	1.029	88	0.807	0.560	1.161	125	0.785	0.549	1.121
Active	186	**0.657**	**0.513**	**0.842**	98	**0.622**	**0.444**	**0.870**	88	**0.681**	**0.470**	**0.987**
Passive	228	**0.746**	**0.586**	**0.949**	121	0.741	0.534	1.028	107	0.738	0.512	1.064
Low strain (ref)	262	1			161	1			101	1		
ISO-strain	154	0.794	0.585	1.078	63	0.889	0.565	1.399	91	0.698	0.447	1.090
Low strain-high support (ref)	177	1			106	1			71	1		

Model 1: adjusted for age, education, smoking, alcohol, physical exercise.

Model 2: model 1 + diabetes heredity, metabolic syndrome, BMI.

Significant values are in bold.

Model 1, adjusted for age, education, smoking, alcohol consumption and physical exercise, showed that active work condition were associated with lower risk for prediabetes compared to the low strain reference group, in the whole population as well as in men. Model 2, which was adjusted for model 1+ diabetes heredity, metabolic syndrome and BMI showed that active and passive work conditions were associated with lower risk for prediabetes among all participants. When analyzing men and women separately, only an active psychosocial work condition was associated with lower risk for prediabetes in fully adjusted model.

Further, similar analyses as mentioned above were performed for iso-strain i.e. the combination of high-strain and low social support with low strain-high support as reference. No increased risk for prediabetes could be observed in the iso-strain group, nor for the whole group or when analyses were performed in men and women separately.

## Discussion

The aim of the study was to investigate whether psychosocial work stressors evaluated with the Job Demand–Control-social support model increases risk for early dysregulated glucose metabolism (prediabetes) in 50–64-year-old men and women. Surprisingly and in contrast to much of previously published data, our analyses could not corroborate that high strain job conditions were associated with increased risk of prediabetes. Interestingly however, the logistic regression analysis performed in the present study showed that an active psychosocial work condition, characterized by high job demands and high job decisions latitude, was associated with a lower risk of prediabetes in men and women separately, as well as among all participants in a regression model adjusted for age, education, smoking, alcohol consumption, physical exercise, diabetes heredity, metabolic syndrome and BMI. To our knowledge, this is the first study showing that both men and women assessing their JDC as active i.e. high job demand in combination with high job decisions latitude, had lower risk for prediabetes. According to the JDC model, an active job condition is often characterized by demanding work situations, but also involve the workers in activities over which have a larger degree of control^19^. An active work situation is also more common in high status jobs^20^, suggesting that a high socioeconomic status could serve as a protective dimension in this group. However, the finding was robust even when adjusting for education and several risk factors associated with low socioeconomic status.

In contrast to previous studies^10,11^, the current analyses do not lend support for the hypothesis that psychosocial work conditions characterized as high job demands and low job decisions latitude i.e. high strain, is associated with dysregulated glucose metabolism in men nor women. Interestingly, this was true despite the fact that the high-strain group showed a more unfavourable lifestyle compared to the low-strain group. The group assessed with high strain work had higher BMI, as well as a greater number of participants with abdominal- and general obesity compared to the low-strain group. Further, participants in the high strain group was more likely to be smoking, to be less physically active, have less alcohol consumption, and a greater probability to have heredity for diabetes in the family. This is much in line with previous studies showing that participants with high strain jobs were more likely to higher odds to be smokers^21,22^, to be non-drinkers and less physically active^23^ or having adverse health behaviours^24^. A theoretical argument is that exposures to job stressors may foster adverse health behaviours as adaptive mechanisms for coping with distress and may simultaneously reduce the odds of making and succeeding at positive health behaviour change^25^. The finding of decreased risk of prediabetes could also reflect a “healthy worker effect,” which mean that only individuals with the resource of strong health will endure job conditions characterized by high demands in combination with low decision latitude^19^. Further, results from previous studies support these findings showing higher odds for inactivity or lower leisure time physical activity among participants working in high-strain jobs compared with those with low-strain jobs^26,27^.

The above analyses were based on categories of glucose intolerance. It is also known that HbA1c is an indicator of work stress and epidemiological studies have observed increased levels of HbA1c in high work stress^9,28,29^. This is the technique used in previous studies of associations between prediabetes and high strain^9,10^. However, in the present study, no differences in HbA1c levels were observed between the various job strain groups. Concentrations of HbA1c are mainly determined by glucose metabolism but previous studies have shown that it is also affected by body fat distribution and smoking^30^.

The Homeostasis Model Assessment of IR (HOMAIR) has proved to be a robust tool for the assessment of insulin resistance (IR) and is the index of IR that is most widely used in large population studies^31^. Previous studies have found increased HOMA-IR levels in health care shift workers and that workers who experienced a decrease in supervisor support had a significantly higher risk for IR compared to workers who´s supervisor support remained stable and high^32,33^. No previous study has studied the relation between high strain work and HOMA-IR. The analyses in this study showed a trend (*p* = 0.201) with the lowest HOMA-IR values in the low strain groups, increasing through group to the highest level in the high strain group.

It is surprising that high strain work is not associated with disturbed glucose homeostasis. There may be situations in the workplace that buffer adverse effects of job strain on health^34^. Physiologically, receiving support has positive immediate and longer-term effects on the cardiovascular-, immune-, and neuroendocrine systems, and hence promotes physiological strengthening that improves individuals' responses to work demands^35^. Social support in form of help and support from co-workers and from immediate superior were measured in the present study. However, the group with high job strain showed significantly less support both from colleagues and from immediate superior compared to the reference group (low strain). In previous literature, the combination of high demand—low control together with low support is known as iso-strain^36^, which has shown to be associated with poor health outcomes. Iso-strain has previously shown to be an independent risk factor for sickness absence due to mental disorders^36^. However, reasons for sickness absence were not measured in the present study, but the high strain group showed increased long-term sickness absence from work compared to the reference group with low strain job condition. Studies suggest that the risk factors of overweight, smoking and inadequate physical activity contribute toward higher sickness absence, even after controlling for health status and workplace factors^37^. As previously mentioned, the high strain group had an accumulation of lifestyle related risk factors such as obesity, low physical activity and increased smoking compared with the reference group, surprisingly, this does not impact on the prevalence for prediabetes.

Individuals with a psychological work condition characterized as low job demands and low job decisions latitude, i.e. passive, showed to have a lower risk for prediabetes, in men, as well as in the total population, in a regression analysis adjusted for age, higher education, smoking, alcohol, physical exercise, diabetes heredity and metabolic syndrome. This was observed despite that the passive group and the reference group, i.e. low strain, showed similar characteristics in general. Passive psychological work conditions have previously shown increased risk of type 2 diabetes in middle-aged US workers, but not in men and women analysed separately^38,39^

To our knowledge, there is a void in the literature regarding iso-strain and prediabetes, and this is the first study to address iso-strain and prediabetes. We were not able to observe an increased risk for prediabetes in the group assessing their psychological work condition with high-strain and low social support. However, there are published papers studies concerning iso-strain and type 2 diabetes^11,40^. Results from one study showed that iso-strain was associated with a two-fold higher risk of type 2 diabetes, in women only, and that this association was consistent through all multivariate-adjusted regression analyses, but this pattern was not observed in men separately, nor in the whole population^40^.

There are both strengths and limitations to the present study. Strengths are; well-defined and reasonably large study groups in the same range of age and living in the same area in Sweden. Limitations are the cross-sectional design which cannot analyze behaviour over a period to time and it does not determine cause and effect. Further limitations are that income, occupational social class, and area deprivation, are not available which poses a threat to the internal validity of the findings. I addition, diet is also an important covariate not available and this may have implications on the findings.

In conclusion, our hypothesis that psychosocial stress defined as high strain based on the Job Demand–Control model increases risk for early dysregulated glucose metabolism could not be supported from the current study. This is especially surprising given the accumulation of life-style related risk factors in this group. Interestingly, our study showed that both men and women assessing their work conditions as active, showed lower risk for dysregulated glucose metabolism.

## Data Availability

The datasets generated and/or analyzed during the current study are not publicly available due to the sensitive nature of the personal data and study materials, they cannot be made freely available but are available from the corresponding author on reasonable request and be arranged following Swedish legislation.
